# TET2 suppresses nasopharyngeal carcinoma progression by inhibiting glycolysis metabolism

**DOI:** 10.1186/s12935-020-01456-9

**Published:** 2020-08-03

**Authors:** Xixia Zhang, Jing Yang, Dong Shi, Zhiwei Cao

**Affiliations:** Department of Otolaryngology Head and Neck Surgery, Shengjing Hospital of China Medical University, No. 36 Sanhao Road, Shenyang, 110004 Liaoning China

**Keywords:** Nasopharyngeal carcinoma, Ten-eleven translocation protein 2, Pyruvate kinase, muscle, Glycolysis

## Abstract

**Background:**

Nasopharyngeal carcinoma (NPC) is a common malignant tumor. Ten-eleven translocation (TET) protein 2 (TET2), an evolutionarily conserved dioxygenases, is reported to be involved in various malignant tumor developments. Here, we aim to investigate the effect of TET2 on NPC progress in vitro and in vivo, and its detailed underlying mechanism.

**Methods:**

Real-time PCR and western blotting were used to determine the expression levels of TET1/2/3 in NPC cell lines. The effects of TET2 on NPC progression were evaluated using CCK8 and invasion assays in vitro. Proteins interacted with TET2 in NPC cells were detected by immunoprecipitation and mass spectrometry. The effects of TET2 or pyruvate kinase, muscle (PKM) on glycolysis in NPC cells were examined by detecting glucose uptake and lactate production. The effects of TET2 on NPC progression were evaluated using xenograft tumor model in vivo.

**Results:**

TET2 expression was decreased in NPC cells, and TET2 overexpression inhibited proliferation and invasion of NPC cells, which is independent on TET2’s catalytic activity. In mechanism, TET2 N-terminal domain interacts with PKM in cytoplasm to prevent PKM dimers from translocating into nucleus, suppressing glycolysis in NPC cells, thereby inhibiting proliferation and invasion of NPC cells. Moreover, using xenograft tumor model, we found that TET2 knockout promoted NPC progression and decreased survival rate. However, administration with the inhibitor of PKM, shikonin, decreased the tumor volume of TET2-cas9 group, and increased the survival rate.

**Conclusion:**

TET2 suppresses NPC development through interacting with PKM to inhibit glycolysis.

## Background

Nasopharyngeal carcinoma (NPC) is characterized by distinct geographical distribution and has a high incidence in southeast Asia and north Africa [1]. Multiple factors, such as Epstein-Barr virus infection, host genetics, and environmental factors contribute to NPC development [2]. Although after radiotherapy and concurrent adjuvant chemotherapy, clinical outcomes of NPC patients have improved, tumor recurrence and distant metastasis are still a tough challenge [3, 4]. Therefore, deeply elucidating the detailed mechanism of NPC development is essential for developing novel therapeutic drugs.

Ten-eleven translocation (TET) protein family, including TET1, TET2, and TET3, are evolutionarily conserved dioxygenases, which regulate gene transcription through DNA demethylation via catalyzing the oxidation of 5-methylcytosine (5-mC) to 5-hydroxymethylcytosine (5-hmC), which is generally considered as active sites for gene transcription [5–7]. Recently, emerging studies report that TET2 mutations are closely associated with many tumorous developments, including acute myelocytic leukemia, chronic myelomonocytic leukemia, angioimmunoblastic T cell lymphoma and mature lymphoid neoplasms [8–10]. Besides, abnormal expression of TET2 is reported to be involved in some tumorous progression and drug resistance. For example, increased expression of TET2 promotes melanoma progression by suppressing tumor-infiltrating myeloid cells [11]. Downregulation of TET2 promotes gastric cancer cisplatin resistance via regulating interleukin-6 expression in the tumor microenvironment [12]. TET2 loss promotes tumorigenesis of breast cancer cells by regulating caspase-4 [13]. Recent study reports that 5-hmC level is decreased in metastatic tissues of nasopharyngeal carcinoma, breast cancer, and colon cancer, compared with non-metastasis tumor tissues, and TET2 is involved in the process of tumor metastasis [14]. However, the detailed molecular mechanism of TET2 in NPC progression is still not fully understood.

Hyperactivity of aerobic glycolysis or the Warburg effect is the hallmark of NPC metabolism [15]. Glycolytic metabolism is essential for the perpetual proliferation of tumor cells, and is tightly controlled by various glycolytic enzymes, of which hexokinase (HK), phosphofructokinase (PFK), and pyruvate kinase (PK) are the three pivotally rate‐limiting enzymes of glycolysis [16–18]. In mammals, the M2 isoform of pyruvate kinase, muscle (PKM2), which is universally expressed during embryogenesis, regeneration, and cancer, is the last step of glycolysis and catalyzes phosphoenolpyruvate into pyruvate [19]. Then, lactate dehydrogenase (LDH) catalyzes pyruvate to lactate [20]. Most of the enzymes involved in glycolysis have been reported to be overexpressed in tumors, such as lung cancer, hepatocellular carcinoma, and NPC [21–23]. Recently, the 5-mC and 5-hmC modification levels of the genes involved in glycolysis have been reported to differ significantly between hepatocellular carcinoma tissues and paired adjacent peritumor tissues [24]. However, whether TET2 is involved in regulating glycolysis during NPC tumorigenesis is completely unclear.

In the current study, we investigated the expression and biological functions of TET2 in NPC cells and found TET2 plays an inhibitory role on NPC progression. Moreover, we demonstrated that TET2 suppresses NPC progression through interacting with PKM to decrease glycolysis in NPC.

## Materials and methods

### Cell culture

The normal nasopharyngeal cell line NP69,obtained from the Chinese Academy of Science (Beijing, China), was cultured in keratinocyte/serum-free medium (Thermo Fisher) supplemented with bovine pituitary extract (BD Biosciences). Human NPC cell lines CNE1, HNE1, 5-8F, and SUNE-1, obtained from Jiwei Biotechnology (Shanghai, China), were cultured in RPMI-1640 medium supplemented with 10% fetal bovine serum (FBS) (Gibco) and 1% penicillin–streptomycin (Gibco). The cells were cultured at 37 °C with 5% CO_2_ in a humidified incubator.

### RNA extraction and qRT-PCR

RNA Extraction and qRT-PCR were performed as previously described [25]. Briefly, cellular RNA was extracted from each sample with the Trizol reagent (Thermo Fisher) and then reverse-transcribed into cDNA using the PrimeScript RT re-agent kit (Takara). QRT-PCR was performed using the 7500 real-time PCR system (Applied Biosystems), with AceQ qPCR SYBR Green Master Mix (Roche). The obtained data were normalized to GAPDH expression levels in each sample. The primers for qRT-PCR were as following: TET1 F: 5′-CGCTACGAAGCACCTCTCTTA-3′, TET1 R: 5′-CTTGCATTGGAACCGAATCATTT-3′; TET2 F: 5′-CCCACAGAGACTTGCACAACAT-3′, TET2 R: 5′-CTGGCTCTGCTAACATCCTGAC-3′; TET3 F: 5′-AGTGTCCGAAAGCCCATTCAG-3′, TET3 R: 5′-GCAAATAGCGCAAGAGAAGGTT-3′; GAPDH F:5′-TCAACAGCAACTCCCACTCTTCCA-3′, and GAPDH R: 5′-ACCCTGTTGCTGTAGCCGTATTCA-3′, respectively.

### siRNAs, plasmids, lentiviruses and drugs

siRNA targeting TET2 (siR-TET2), siRNA targeting PKM (siR-PKM), and scrambled siRNA (siR-NC) were purchased from GenePharma (Shanghai, China). cDNA encoding full-length human TET2 (2002 amino acids) was cloned into Flag-tagged vector (pCMV-Flag), and the truncated TET2 proteins [N-terminal (residues 1-1127 amino acids), C1-terminal (residues 1128–2002 amino acids), C2-terminal (residues 1399–2002 amino acids), double-strand beta helix domain (DHBS, residues 1868–2002 amino acids)] were sub-cloned into pCMV-Flag vector; All constructs were confirmed by DNA sequencing. siRNAs (50 nM) or plasmids (2 µg/ml) were transfected using Lipofectamine 3000 (Thermo Fisher Scientific, USA), as previously described [25]. Lentivirus expressing wild type TET2 (Lenti-TET2) or catalytic inactive mutant (H1367Y and D1369A) [26] (Lenti-TET2Δ) were constructed and purchased from GenePharma (Shanghai, China). The TET2 inhibitor, Dimethyloxallyl glycine (DMOG, 10 µM) and Bobcat339 (BC339, 80 µM), the glucose analog 2-deoxyglucose (2-DG, 2 mM), and ATP (5 mM) were purchased from Selleck (Selleck, USA). The PKM inhibitor, shikonin, was purchased from Sigma (Sigma, USA).

### Invasion assay

Invasion assay was performed, as previously described [25]. After transfection, CNE1 or SUNE1 cells (2 × 10^4^) were seeded in the upper chamber (8 mm pore; Millipore) containing FBS-free medium with the Matrigel-coated membrane. Subsequently, 10% FBS was added to the lower chamber as a chemoattractant. After 24 h, cells were fixed with 3% paraformaldehyde and stained with 0.1% crystal violet, and counted using the inverted microscope.

### Cell proliferation assay

Cell proliferation assay was performed, as previously described [25]. After transfection, CNE1 or SUNE1 cells were cultured in a 96-well plate (3000 cells/well). Triplicate wells were measured in each group. Cell viability was determined every 24 h. The plate was incubated at 37 °C for 2 h after each well was added with 10 μl CCK-8 solution (Dojindo, Japan). Then the spectrophotometric absorbance was measured at 450 nm for each sample.

### Western blot

After treatments, NPC cells were washed with PBS and then lysed with cell lysis buffer (CST, USA). Whole cell lysates were centrifuged at 14,000*g* for 5 min at 4 °C to remove cell debris, and the supernatants were subjected to SDS-PAGE and immunoblotting, as previously described [25]. The following antibodies used in this study were purchased from Abcam (USA): rabbit anti-TET1 (ab191698), rabbit anti-TET2 (ab94580), rabbit anti-TET3 (ab139311), mouse anti-GAPDH (ab8245), rabbit anti-flag (ab1162), rabbit anti-PKM antibody (ab131021), and rabbit anti-Histone H3 antibody (ab176842).

### Immunoprecipitation and mass spectrometry

Immunoprecipitation was performed as previously described [25]. Briefly, after transfection, CNE1 or SUNE1 cells were collected and lysed with lysis buffer (50 mM Tris–HCl and 0.1% Np-40, pH 7.4). After centrifugation, the cell lysates were precleared with protein A/G agarose beads (Santa Cruz Biotechnology, USA) and IgG. Subsequently, the samples were incubated with the anti-flag/TET2 antibody or IgG, and protein A/G agarose beads overnight at 4 °C with continuous rotation. The beads were then washed three times in lysis buffer, and the immunoprecipitation complexes were eluted from protein A/G agarose beads by heating at 100 °C for 5 min. Following centrifugation, the samples were subjected to SDS-PAGE electrophoresis. Then, the gel was stained with the Fast Silver Stain Kit (Beyotime, Shanghai, China), and analyzed by reverse-phase liquid chromatography coupled with tandem mass spectrometry in Huada protein research and development center (Beijing, China). Mass spectrometry peptide sequences and protein identity were determined by matching fragmentation patterns in protein databases using the Mascot software program (Matrix Science, MA). Enzyme specificity was set to partially tryptic with two missed cleavages. Mass tolerance was set to 20 ppm for precursor ions and fragment ions. The database searched was Swiss-Prot (Homo sapiens). Spectral matches were filtered to contain the false-discovery rate to less than 1% at the peptide level using the target-decoy method by Huada protein research and development center (Beijing, China).

### Nuclear and cytoplasmic extraction

cellular nuclear and cytoplasmic fractions were extracted using NE-PER™ Nuclear and Cytoplasmic Extraction Reagents (Thermo Scientific, USA), according to the manufacture’s instruction.

### Dot-blot assay

Dot-blot assay was performed following previously reported [27]. Briefly, CNE1 cells were cultured in 6-well plate and treated with the TET2 inhibitor, Dimethyloxallyl glycine (DMOG, 100 µM, Selleck) for 72 h, or Bobcat339 (BC339, 80 μM, Selleck) for 24 h. DMSO treatment was used as control. Cellular genomic DNA (500 ng) was extracted by the Wizard Genomic DNA Purification Kit (Promega). DNA samples were diluted in TE buffer, denatured in 0.4 M NaOH/10 mM EDTA for 10 min at 95 °C, neutralized with equal volume of 2 M NH_4_OAc (pH 7.0), and spotted on a nitrocellulose membrane (pre-wetted in 1 M NH4OAc,pH 7.0) in two-fold serial dilutions using a Bio-Dot Apparatus Assembly (Bio-Rad). The blotted membrane was rinsed briefly in 2 × SSC, air-dried, baked at 80 °C for two hours, blocked in 5% non-fat milk for 1 h at room temperature, and incubated with anti-5hmC (ab106918, Abcam) at 4 °C overnight. After washing three times, the membrane was incubated with HRP-conjugated anti-rabbit IgG secondary antibody, treated with ECL substrate and developed using film.

### CRISPR knockout cell line construction

For generation of stable cell pools with TET2 knockout, sgRNA designed (http://crisper.mit.edu/) targeting TET2 was synthesized and cloned into cas9/gRNA plasmids. CNE1 cells were transfected with cas9/gRNA plasmids and screened by puromycin (Amresco, OH, USA). TET2 knockout cell clones were identified by western blotting.

### mRNA stability

To measure the effects of TET2 knockout on PKM mRNA stability, normal CNE1 cells and TET2 knockout-CNE1 (TET2-cas9) cells were treated with 5 μg/ml actinomycin D (Act-D, Sigma, USA) for the indicated times. Then, cells were collected, and RNA was isolated for qRT-PCR. The mRNA half-life (t1/2) of PKM was calculated using ln2/slope, and GAPDH was used for normalization.

### Chromatin immunoprecipitation (CHIP)

CHIP was performed using an EZ-Magna ChIP Chromatin Immunoprecipitation Kit (Millipore, USA), as previously described [25]. Briefly, CNE1cells were lysed, and the chromatin was mainly fragmented ranged from 300 bp to 900 bp by prior digestion with micrococcal nuclease and later ultrasonication. DNA/protein complexes were precipitated by overnight incubation with 4 μg antibodies against histone H3 tri-methylated lysine 4 (H3K4me3, ab185637, Abcam), and then incubated with magnetic protein A/G agarose beads for 2 h. After reversal of protein-DNA cross-links, the DNA was purified and then analyzed by qPCR. The primers for ChIP were as following: PKM promoter 0-0.5 k F: 5′-GGGCCAGACTGTTTCCTCTC-3′ and R: 5′-CTTTCTCCCAGGGCGACTTT-3′; PKM promoter 0.5–1 k F: 5′-GGAAGGAGAGAAGCTGGGGA-3′ and R: 5′-TCCGGCTTAAAGCGGTCATC-3′; PKM promoter 1–1.5 k F: 5′-GCTTTTCCTCTCCCCTGACC-3′ and R: 5′-TCAAAGGCAGGGAAGCAGAG-3′; PKM promoter 1.5 k–2 k F: 5′-CTCTTTTCTGCTGGGGAGGG-3′ and R: 5′-GACACCACCATAGCTCCCAC-3′.

### Detection of glucose uptake

Glucose uptake was determined by a Glucose Colorimetric Assay Kit (Biovision, CA), according to the according to the manufacture’s instruction. CNE1 or TET2-knockouted CNE1 cells were cultured into 24-well plates and transfected with siR-NC or siR-PKM for 24 h. Then, supernatant of cell culture medium was collected for detection of glucose uptake. Three independent experiments were carried out for each assay.

### Detection of lactate production

Lactate production was determined by a Lactate Colorimetric Assay Kit (Biovision, CA), according to the according to the manufacture’s instruction. CNE1 cells were cultured into 24-well plates and transfected with pcmv-flag-TET2 or its mutants for 24 h. Then, supernatant of cell culture medium was collected for detection of lactate production. Three independent experiments were carried out for each assay.

### Detection of phosphoenolpyruvate (PEP) production

PEP production was determined by a PEP Colorimetric Assay Kit (Biovision, CA), according to the according to the manufacture’s instruction. CNE1 or TET2-knockouted CNE1 cells were cultured into 24-well plates and transfected with siR-NC or siR-PKM for 24 h. Then, supernatant of cell culture medium was collected for detection of PEP production. Three independent experiments were carried out for each assay.

### Detection of pH value

pH value was determined by a Intracellular pH Assay kit (Abcam, CA), according to the according to the manufacture’s instruction. CNE1 or TET2-knockouted CNE1 cells were cultured into 24-well plates and transfected with siR-NC or siR-PKM for 24 h. Then, supernatant of cell culture medium was collected for detection of PH value. Three independent experiments were carried out for each assay.

### Xenograft tumor model

One hundred and twenty 4-week-old male BALB/c nude mice were divided into 6 groups randomly. Each group was composed of twenty mice. For exploring the effects of TET2 knockout on tumor development, nude mice were subcutaneous injected with 2 × 10^6^ CNE1 cells (control) or TET2 knockouted-CNE1 cells (TET-cas9). Tumor volume (length × width^2^ × 0.5) was recorded every 5 days post-injection. Survival rate of nude mice were recorded everyday as long as 60 days. For exploring the effects of PKM inhibitor, Shikonin, on NPC progress, after the xenografts were established, the tumor-bearing mice (wild type CNE1 group or TET-cas9 group) were intravenous injection with shikonin (5 mg/kg, Sigma-Aldrich, USA) for every 2 days, or DMSO. Then tumor volume and survival rate of tumor-bearing mice were recorded. All animal studies were approved by the Animal Ethics Committee of China Medical University and experiments were conducted according to the National Institutes of Health Guide for the Care and Use of Laboratory Animals.

### Statistical analysis

All data are presented as mean ± standard deviations (SD). Statistical comparison was performed using Student’s t-test and P < 0.05 was considered statistically significant.

## Results

### TET2 inhibits proliferation and invasion of NPC cells

**Fig. 1:**
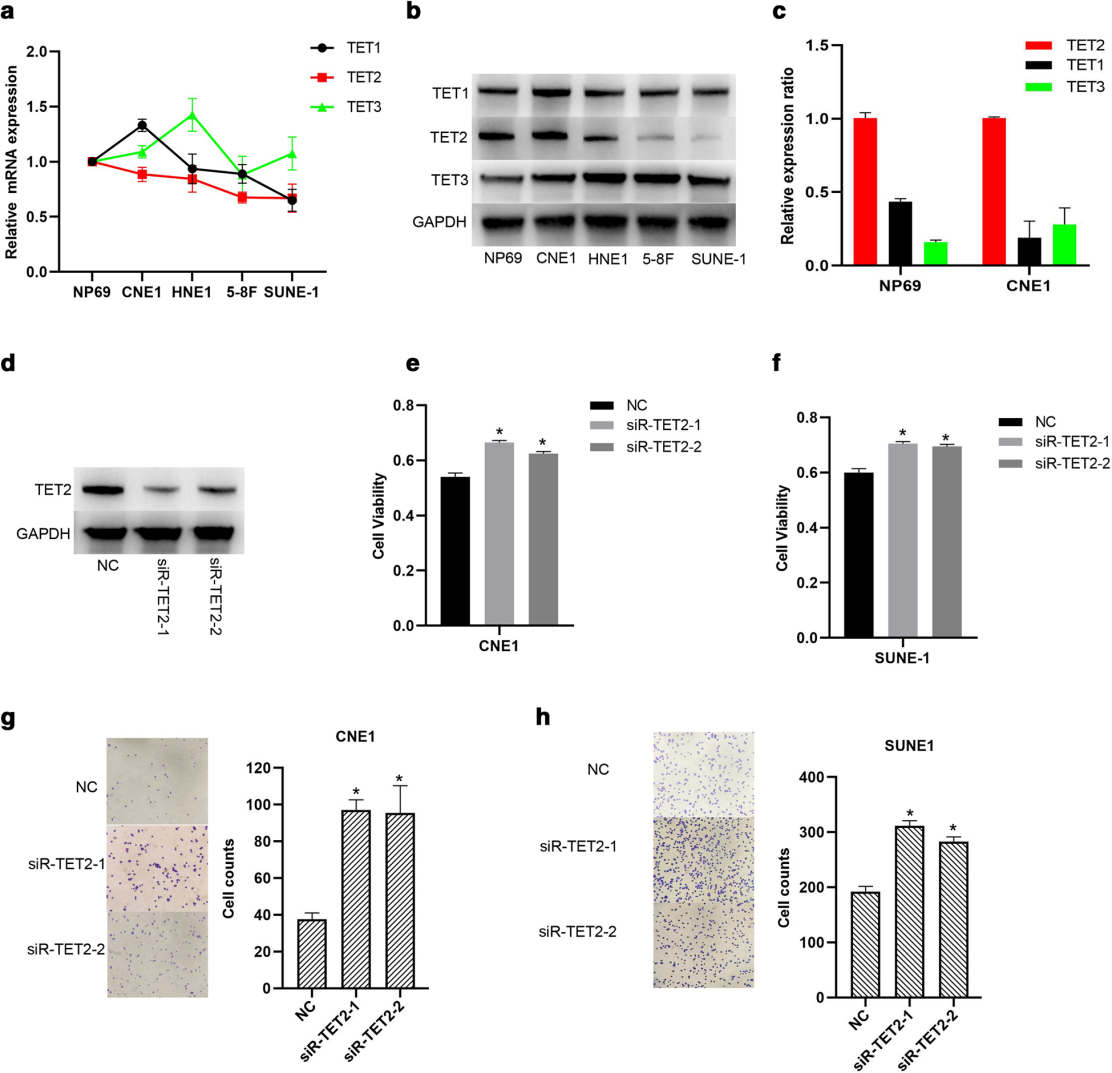
TET2 inhibits proliferation and invasion of NPC cells. **a** TET1/2/3 mRNA expression levels were examined in several NPC and the immortalized nasopharyngeal cell line NP69 by qRT-PCR. **b** TET1/2/3 protein expression levels were examined in the indicated cells by western blotting. **c** Relative mRNA expression level of TET1/3 to TET2. **d** The knockdown efficiency of siR-TET2 was examined by Western blotting in CNE1 cells. Cell viability of TET2-knockdown CNE1 (**e**) and SUNE1 (**f**) cells were examined. Cell invasive ability of TET2-knockdown CNE1 (**g**) and SUNE1 (**h**) were examined. Data are represented as mean ± SD (n = 3; *represents P < 0.05)

TET protein family are usually considered as tumor suppressors by promoting locus-specific reversal of DNA methylation [13, 28, 29], however, the detailed molecular mechanisms of TET protein family regulating NPC progress are still unclear. In this study, we first examined the mRNA expression levels of TET family genes in NPC cell lines. As shown in Fig. 1a, TET1 and TET3 mRNAs were variously expressed in NPC cells (CNE1, HNE1, 5-8F and SUNE-1) and the immortalized nasopharyngeal cell line NP69. Interestingly, TET2 mRNA expression levels of NPC cell lines were relatively lower that of NP69 cells, and TET2 mRNA expression levels in 5-8F and SUNE-1 cells were lower those in the poorly differentiated CNE1, and HNE1 cells. Consistently, TET2 protein expression levels in HNE1, 5-8F and SUNE-1 cells were also lower that in NP69 cells (Fig. 1b). QPCR analysis of mRNA expression in NP69 and CNE1 cells also showed that TET2 mRNA abundance was higher than TET1 and TET3 (Fig. 1c). Given that TET2 expression level of NPC cells was significantly lower than that of immortalized nasopharyngeal cells, we further explored the roles of TET2 on NPC progress in the following studies. The results showed that TET2 knockdown significantly promoted CNE1 and SUNE-1 cell proliferation (Fig. 1d–f ). Besides, TET2 knockdown also significantly promoted NPC cell invasion (Fig. 1g, h). With above evidence, we conclude that TET2 inhibited proliferation and invasion of NPC cells.

### TET2 inhibiting proliferation and invasion of NPC cells is independent on TET2’s catalytic activity

**Fig. 2:**
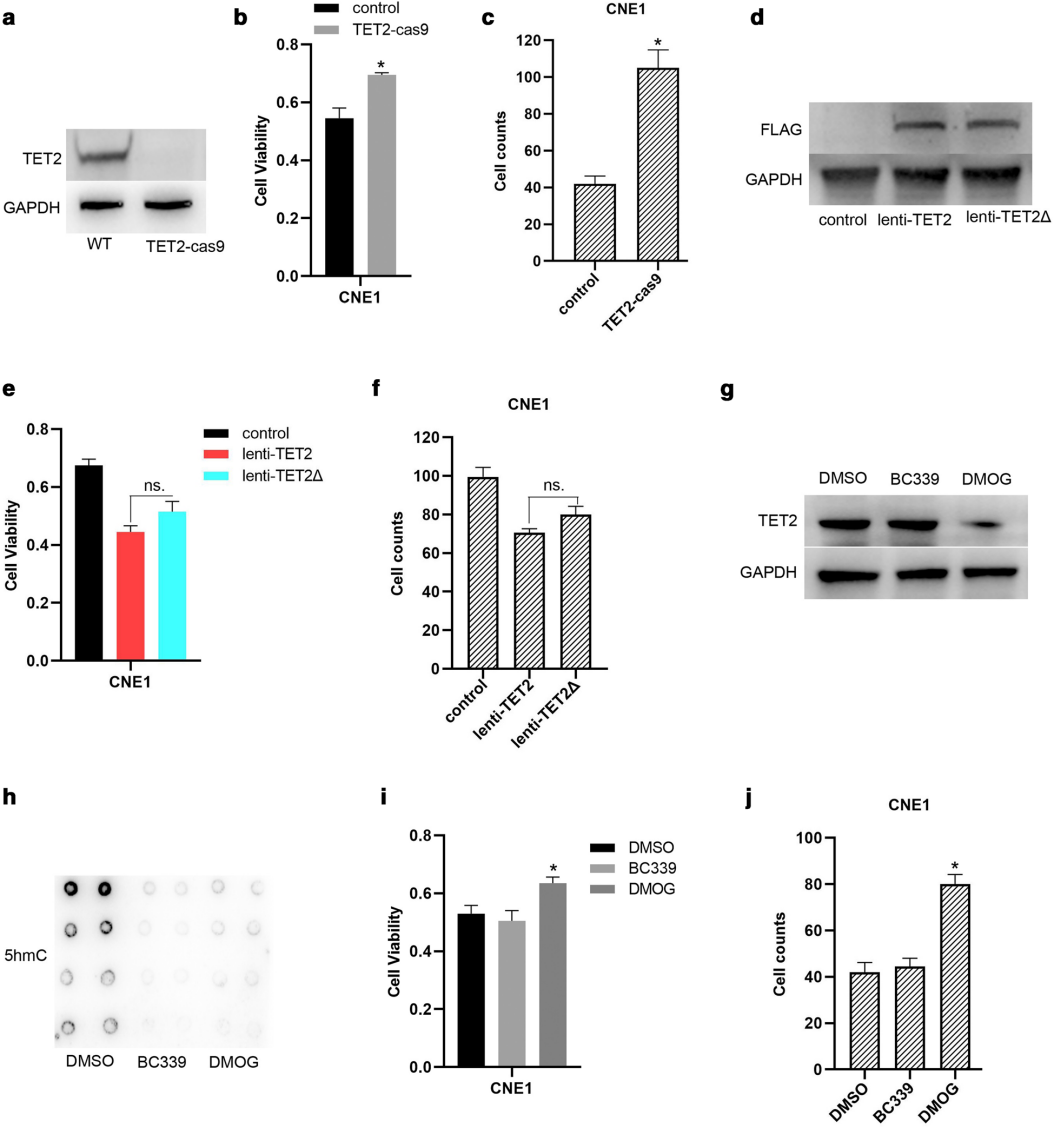
TET2 inhibiting proliferation and invasion of NPC cells is independent on TET2’s catalytic activity. **a** TET2 expression in wild type (WT) CNE1 cells or TET2 knockout-(TET2-cas9) CNE1 cells was examined by western blotting. Cell viability (**b**) and cell invasive ability (**c**) of TET2-knockdown CNE1 cells were examined. **d** TET2 expression in CNE1 cells (control), CNE1 cells infected with lentivirus expressing flag-tagged TET2 (lenti-TET2), or CNE1 cells infected with flag-tagged TET2 mutant with deficiency of catalytic activity, was examined by western blotting. Cell viability (**e**) and cell invasive ability (**f**) of the indicated cells were examined. **g** TET2 expression in DMSO (control), TET2 inhibitor Bobcat339 (BC339, 80 μM), or dimethyloxallyl glycine (DMOG, 10 μM), treated CNE1 cells were examined. **h** 5hmc levels of the indicated cellular genome was detected by dot-blot. Cell viability (**i**) and cell invasive ability (**j**) of the indicated cells were examined. Data are represented as mean ± SD (n = 3; *represents P < 0.05)

To examine whether the demethylated catalytic activity is involved in TET2 promoting proliferation and invasion of NPC cells, we generated TET2-knockout CNE1 cells by CRISPR/Cas9 method (Fig. 2a). Consistent with the results of Fig. 1e, g , TET2 knockout significantly promoted proliferation and invasion of CNE1 cells (Fig. 2b, c). Furthermore, TET2 overexpression mediated by lentivirus significantly inhibited proliferation and invasion of CNE1 cells (Fig. 2d–f ). Besides, overexpression of TET2 mutant with deficiency of catalytic activity also markedly inhibited proliferation and invasion of CNE1 cells, which was comparable to the effects of wild type TET2 overexpression (Fig. 2e, f). Moreover, to confirm the results of Fig. 2e, f, Bobcat339 (BC339), which is a promising cytosine-based TET2 inhibitor [30], and dimethyloxallyl glycine (DMOG), a non–specific 2–OG–dependent dioxygenase inhibitor, which not only inhibits TET2’s catalytic activity but also decreased TET2 expression [31], were used to inhibit TET2’s catalytic activity. As shown in Fig. 2g, h, compared to DMSO treatment, BC339 or DMOG treatment significantly suppressed 5-hmC level of genome in CNE1 cells. Consistent with the results of Fig. 2e, f, BC339 treatment did not affect proliferation and invasion of CNE1 cells, but DMOG treatment markedly increased (Fig. 2i, j). Collectively, our findings indicate that TET2 promoting proliferation and invasion of NPC cells is independent on TET2’s catalytic activity.

### TET2-PKM interaction is required for TET2 inhibiting proliferation and invasion of NPC cells

**Fig. 3:**
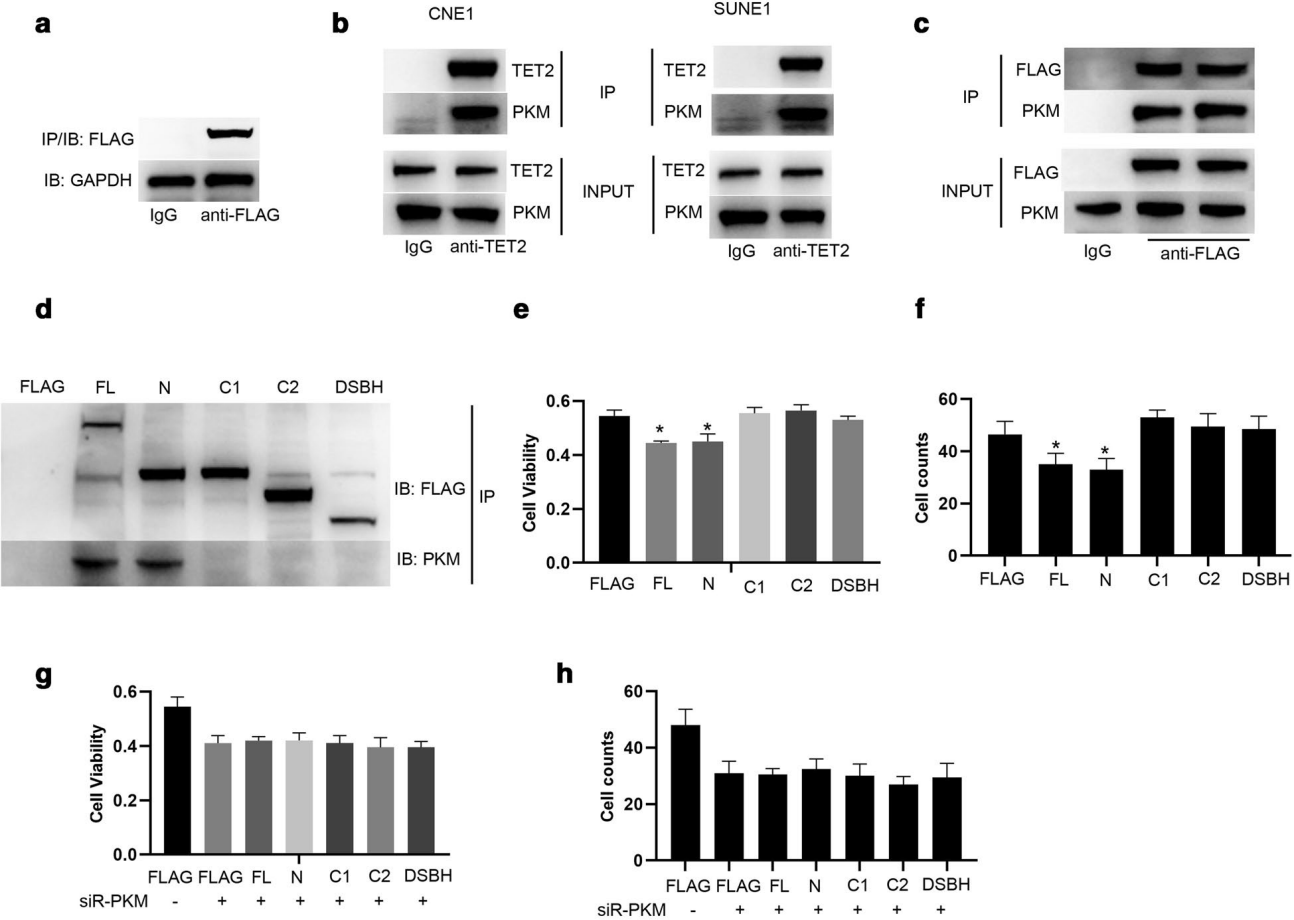
TET2-PKM interaction is required for TET2 inhibiting proliferation and invasion of NPC cells. **a** Flag-TET2 was immunoprecipitated and examined by western blotting. **b** Endogenous TET2 interacting with PKM was detected by protein immunoprecipitation in CNE1 and SUNE1 cells. **c** Exogenous TET2 interacting with PKM was detected by protein immunoprecipitation in CNE1 cells. **d** Full length (FL) TET2 or different truncations of TET2 interacting with PKM was detected by protein immunoprecipitation in CNE1 cells (N: N-terminal TET2, residues 1–1127 amino acids; C1: C1-terminal TET2, residues 1128–2002 amino acids; C2: C2-terminal TET2, residues 1399–2002 amino acids; DSBH: double-strand beta helix domain of TET2, residues 1868–2002 amino acids). Cell viability (**e**) and cell invasive ability (**f**) of CNE1 cells transfected with the indicated plasmids were examined. Cell viability (**g**) and cell invasive ability (**h**) of CNE1 cells transfected with the indicated plasmids and siR-PKM were examined. Data are represented as mean ± SD (n = 3; *represents P < 0.05)

To investigate the molecular mechanism underlying TET2 promoting proliferation and invasion of NPC cells, the proteins interacted with ectopically expressed TET2 (Flag-tagged TET2) in CNE1 cells were immunoprecipitated by anti-Flag antibody and then analyzed by mass spectrometry (Fig. 3a). Among the identified proteins by mass spectrometry (Additional file 1: Table S1), we were interested in pyruvate kinase, muscle (PKM), which is responsible for catalyzing phosphoenolpyruvate into pyruvate in the last step of glycolysis and is highly expressed in bladder cancer and hepatocellular carcinoma [32, 33]. Subsequently, TET2 interacting with PKM was demonstrated through protein immunoprecipitation in CNE1 and SUNE1 cells (Fig. 3b). Furthermore, ectopically expressed TET2 also interacted with PKM (Fig. 3c).

To investigate the detailed domain of TET2 responsible for binding PKM, different TET2 truncations were constructed. We found that N-terminal domain (residues 1–1127 amino acids) of TET2 is essential for interacting with PKM (Fig. 3d). Moreover, we found that overexpression of full-length TET2 or N-terminal domain of TET2, but not C1-terminal (residues 1128–2002 amino acids), C2-terminal (residues 1399–2002 amino acids) or double-strand beta helix domain (DSBH, residues 1868–2002 amino acids) of TET2, significantly inhibits proliferation and invasion of CNE1 cells (Fig. 3e, f). Whereas, even though overexpression of full-length TET2 or truncated TET2, knockdown of PKM inhibited proliferation and invasion of CNE1 cells (Fig. 3g, h). Taken together, our data demonstrate that TET2-PKM interaction is required for TET2 inhibiting proliferation and invasion of NPC cells.

### TET2 interacts with PKM in cytoplasm to prevent PKM dimer formation

**Fig. 4:**
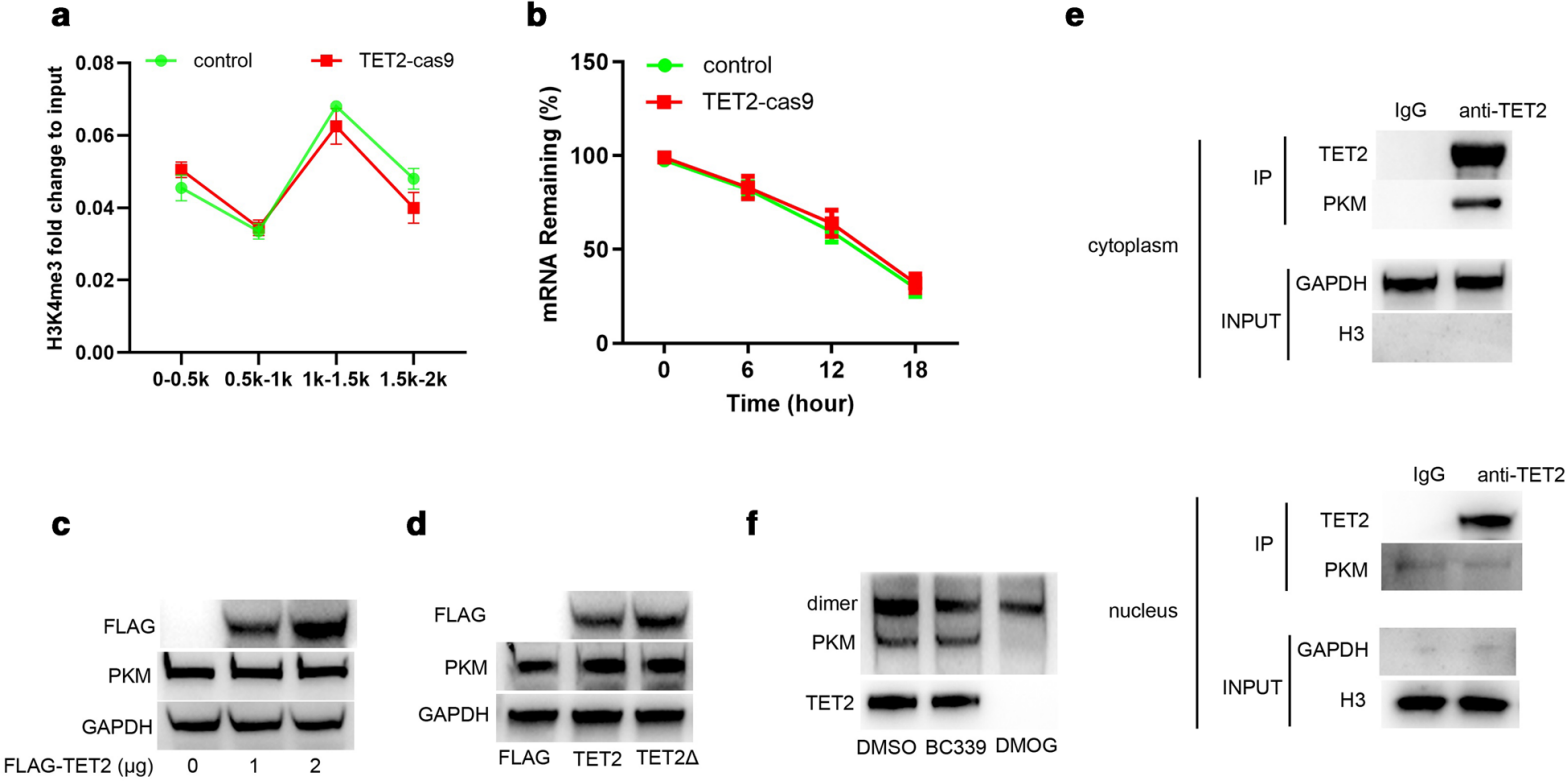
TET2 interacts with PKM in cytoplasm to prevent PKM dimers formation. **a** H3K4me3 modification on the indicated promoter regions of PKM gene was examined by CHIP in CNE cells (control) or TET2-knockout (TET2-cas9) cells. **b** Half-life time of PKM mRNA was examined in CNE cells (control) or TET2-knockout (TET2-cas9) cells. **c** PKM protein expression was examined in CNE1 cells transfected with pcmv-flag-TET2. **d** PKM protein expression was examined in CNE1 cells transfected with pcmv-flag-TET2 or pcmv-flag-TET2Δ with deficiency of catalytic activity. **e** TET2 interacting with PKM was examined in the cytoplasm or nuclear of CNE1 cells. f PKM dimer was examined in DMSO or TET2-inhibitor treated-CNE1 cells. Data are represented as mean ± SD (n = 3; *represents P < 0.05)

Given that TET2-mediated DNA demethylation acts an essential role in the de novo establishment and maintenance of histone H3K4 trimethylation (H3K4me3)/H3K27me3 bivalent domains underlying methylated DNA CpG islands [34], and H3K4me3 is generally related to active transcription [35], we further examined whether TET2 interacting with PKM affected PKM gene expression in NPC cells. The result of chromatin immunoprecipitation (CHIP) assay showed that TET2 knockout did not affect H3K4me3 modification on PKM gene promoter (Fig. 4a). Besides, TET2 knockout also did not affect PKM mRNA stability (Fig. 4b). Subsequently, we further investigated whether TET2 affected PKM protein expression. As shown in Fig. 4c, d, ectopically overexpressed wild type TET2 or TET2 mutant with deficiency of catalytic activity did not affect PKM protein expression. Recent study reports PKM dimer regulates the rate-limiting step of glycolysis by transforming glucose metabolism to lactate production [36]. We subsequently analyzed the location of TET2 and PKM interaction. As shown in Fig. 4e, PKM interacted with TET2 primarily distributed in the cytoplasm. In addition, inhibition of the catalytic activity of TET2 by BC339 did not affect PKM dimer formation, but DMOG treatment significantly decreased PKM dimer (Fig. 4f). Collectively, these results indicate that TET2 interacts with PKM in cytoplasm to prevent PKM dimer formation.

### TET2 interacting with PKM inhibits proliferation and invasion of NPC cells by suppressing glycolysis

**Fig. 5:**
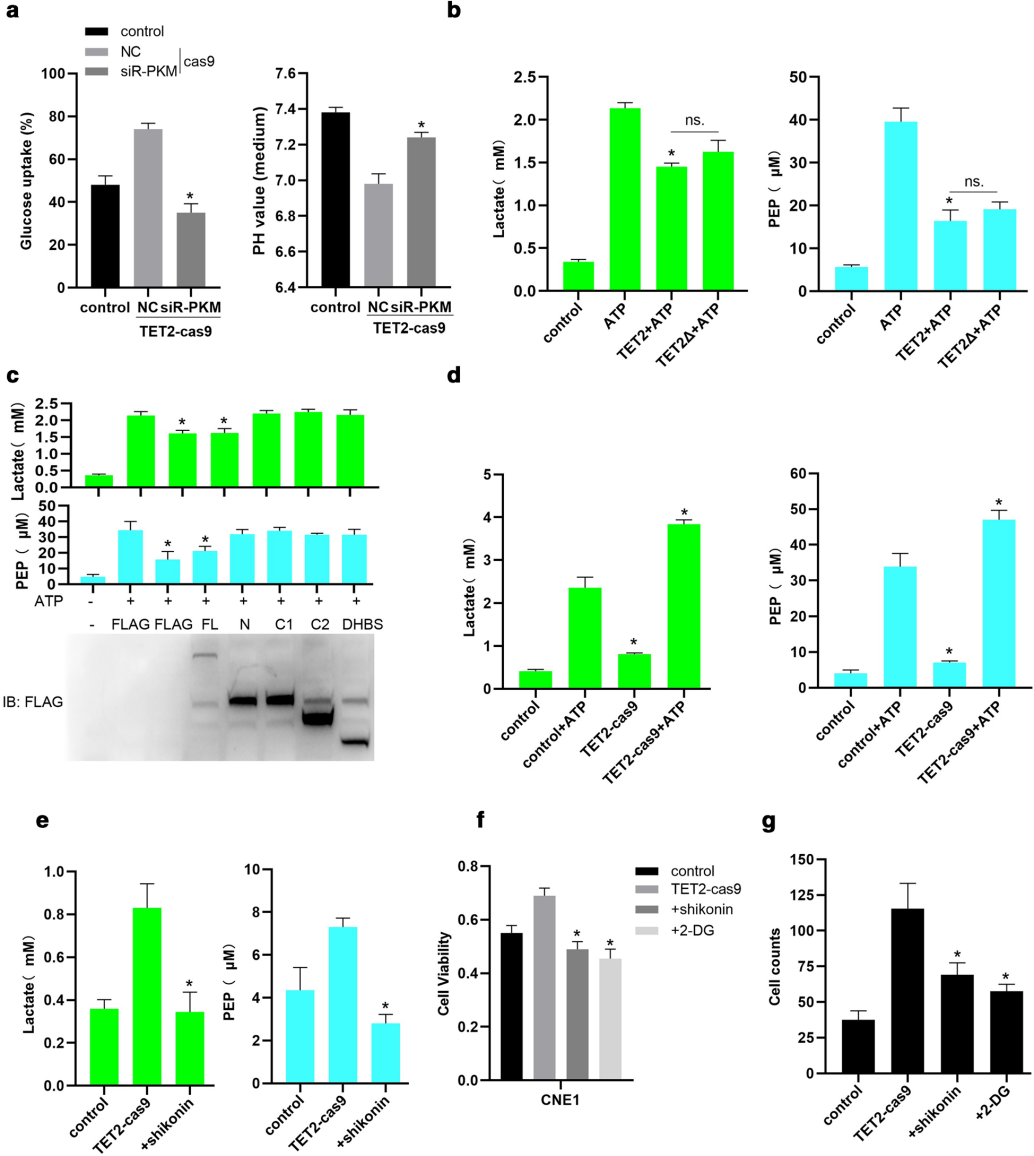
TET2 interacting with PKM inhibits proliferation and invasion of NPC cells by suppressing glycolysis. **a** Glucose uptake or intracellular PH value in CNE1 cells (control) or TET2-knockout CNE1 cells (TET2-cas9) transfected with siR-NC (NC) or siR-PKM. **b** Lactate or PEP production in CNE1 cells (control) or CNE1 cells transfected with pcmv-flag-TET2 or pcmv-flag-TET2Δ with deficiency of catalytic activity, and treated with ATP (100 μmol/L) for 24 h was examined. **c** Lactate or PEP production in CNE1 cells (control) or CNE1 cells transfected with the indicated plasmids and treated with ATP (100 μmol/L) for 24 h was examined. **d** Lactate or PEP production in CNE1 cells (control) or TET2-knockout CNE1 cells (TET2-cas9) treated with ATP (5 mM) for 24 h was examined. **e** Lactate or PEP production in CNE1 cells (control) or TET2-knockout CNE1 cells (TET2-cas9) treated with PKM inhibitor, shikonin (10 μmol/L) for 24 h was examined. Cell viability (**f**) and cell invasive ability (**g**) of CNE1 cells (control) or TET2-knockout CNE1 cells (TET2-cas9) treated with the PKM inhibitor shikonin (10 μmol/L) or the glucose analog 2-deoxyglucose (2-DG, 5 mM) for 24 h was examined. Data are represented as mean ± SD (n = 3; *represents P < 0.05)

Whether interaction of TET2 and PKM inhibits PKM dimer-mediated lactate production? Given that PKM is a rate-controlling enzyme of the glycolytic cascade that catalyzes phosphoenol pyruvate (PEP) into pyruvate, which is then catalyzed into lactate by LDH [19, 20]. Next, we examined the glycolysis level in NPC cells. As shown in Fig. 5a, TET2 knockout significantly promoted glucose uptake, and decreased intracellular PH value. Whereas, PKM knockdown markedly reversed the effects of TET2 knockout on glucose uptake and intracellular PH in CNE1 cells (Fig. 5a). Furthermore, overexpression of wild type TET2 or TET2 mutant with deficiency of catalytic activity significantly suppressed ATP-induced increase in Lactate and PEP production (Fig. 5b). Consistent with that N-terminal domain of TET2 significantly inhibits proliferation and invasion of CNE1 cells (Fig. 3e, f), we also found that TET2 N-terminal domain, but not C1-terminal, C2-terminal or DSBH domain, significantly inhibited ATP-induced Lactate and PEP production (Fig. 5c). In contrast, TET2 knockout not only increased basal productions of Lactate and PEP, but also significantly promoted ATP-induced Lactate and PEP production (Fig. 5d). Moreover, treatment of shikonin (a specific inhibitor of PKM) markedly suppressed TET2 knockout-induced productions of Lactate and PEP (Fig. 5e), suggesting that TET2 inhibiting glycolysis in NPC cells depends on PKM. In addition, we found that treatment with shikonin or 2-deoxy-d-glucose (2-DG, a well-known inhibitor of glycolysis) significantly suppressed TET2 knockout-induced proliferation and invasion of NPC cells (Fig. 5f, g). Collectively, these results indicate that TET2 interacting with PKM inhibits proliferation and invasion of NPC cells by suppressing glycolysis.

### TET2 inhibiting NPC progression in vivo depends on PKM

**Fig. 6:**
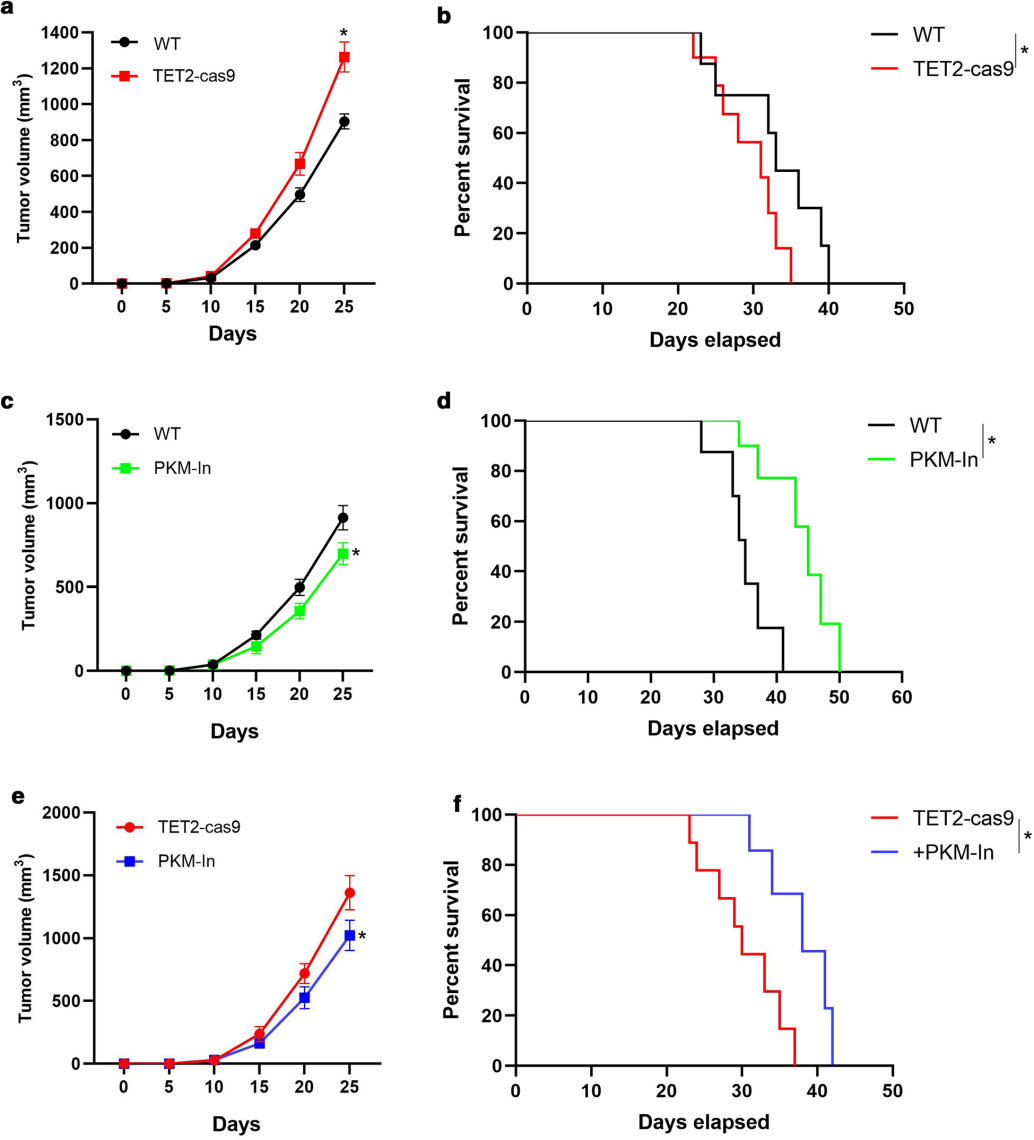
TET2 inhibiting NPC progress in vivo depends on PKM. **a** The tumor volume of nude mice with established subcutaneous TET2-WT (control) and TET2-knockout (TET2-cas9) xenografts was recorded. **b** The survival curve of nude mice with established subcutaneous TET2-WT (control) and TET2-knockout (TET2-cas9) xenografts was calculated. **c** Nude mice with established subcutaneous TET2-WT xenografts were treated with the PKM inhibitor (PKM-In), shikonin (5 mg/kg) or PBS for every 2 days. The animals were maintained for post-treatment observation. The tumor volume of nude mice was recorded. **d** The survival curve of the indicated nude mice was calculated. **e** Nude mice with established subcutaneous with TET2-knockout (TET2-cas9) xenografts were treated with the PKM inhibitor (PKM-In), shikonin (5 mg/kg) or PBS for every 2 days. The animals were maintained for post-treatment observation. The tumor volume of nude mice was recorded. **d** The survival curve of the indicated nude mice was calculated. Data are represented as mean ± SD (n = 3; *represents P < 0.05)

To provide further support for the function of TET2-PKM interaction on regulating NPC progress, we subcutaneously injected wild type CNE1 cells (as control), or TET2-knockout (TET2-cas9) cells in nude mice and found that tumor volume increased in TET2-cas9 group, compared with control group (Fig. 6a). Meanwhile, the survival rate of TET2-cas9 group was lower than that of control group (Fig. 6b). Furthermore, we examined the effect of shikonin on tumor growth in vivo. After the xenografts were established, the tumor-bearing mice were administered with shikonin (5 mg/kg) via intravenous injection. As shown in Fig. 6c, shikonin treatment inhibited NPC growth and increased the survival rate, compared to PBS (control) (Fig. 6c, d). Moreover, administration with shikonin decreased the tumor volume of TET2-cas9 group, and increased the survival rate, compared to TET2-cas9 group (Fig. 6e, f). Collectively, these results indicate that TET2 inhibiting NPC progress in vivo depends on PKM.

## Discussion

NPC is still a serious health problem with remarkable ethnic and geographic distribution in southern China and Southeast Asia [1]. Recently, epigenetic abnormalities are reported to play important roles in tumor development [37], and TET protein family, which demethylates DNA via catalyzing 5-mC to 5-hmC, are found to be participated in several tumor pathogenesis [8–14]. However, the roles of TET family in NPC progression are still not well known. In this study, we find that TET2 protein, but not TET1/3, is evidently lower expressed in NPC cells, and overexpression of TET2 inhibits NPC development in vivo and in vitro. In mechanism, TET2 interacts with PKM to prevent PKM dimers from translocating into nucleus, thereby suppressing glycolysis and NPC development.

TET2, a DNA methylcytosine dioxygenase, functions as a tumor suppressor in hematopoietic malignancies [8–10]. Many studies report that demethylase activity of TET2 is essential for its tumor inhibitory effect [10–13]. Interestingly, we found that the suppressing effect of TET2 on NPC progression is partly independent on its enzymatic activity. Overexpression of TET2 mutant with deficiency of catalytic activity markedly inhibits proliferation and invasion of CNE1 cells, which is comparable to the effects of TET2 overexpression. Through immunoprecipitation and mass spectrometry analysis, we found that TET2 specifically interacts with PKM to slow down lactate production, which is essential for tumorigenesis [36]. PKM acts as a pivotal role in glucose metabolism, PKM1 and PKM2 are formed by alternative splicing from the same single mRNA transcript [18]. PKM1 was expressed mainly in matured brain tissues and skeletal muscle, while PKM2 was widely expressed in embryonic tissues [38]. During tumor progression, PKM1 mRNA was substituted gradually by PKM2 mRNA transcript to reprogram glycolysis in tumor and PKM2 protein expressed increasingly [18, 39]. Combined with previous study [15], we speculated TET2 interacts with the PKM2 isoform in nasopharyngeal carcinoma and inhibits PKM2-mediated glycolysis. While PKM1 isoform differs from PKM2 on 389-433 amino acids sequence, whether TET2 interacts with PKM1 and the relative expression ratio of PKM1 and PKM2 in different nasopharyngeal carcinoma cell lines need to be clarified in future.

PKM2 functions by forming highly active enzymatic tetramers, while PKM2 dimer is nearly non-enzymatic activity but possesses transcriptional activity [40]. The ratio of tetrameric form and dimeric form determines whether glucose carbons are channeled to be utilized for glycolytic ATP production or transcriptional activation [41]. In our study, TET2 interacts with PKM suppressing PKM dimer formation. Downregulation of TET2 promotes PKM dimer formation and lactate production, which seems contradictory. Since PKM2 tetramer was constructed from dimer, we speculated whether enhancing the formation of PKM2 dimer induced by TET2 knockdown also promotes PKM2 tetramer, thereby promoting lactate production in NPC. In addition, whether TET2-mediated transcriptional activity deficiency in PKM2 dimer affects NPC invasion also needs to be investigated in our following studies.

Besides PKM2, some other proteins potentially interacted with TET2 in NPC cells, such as PRKAB2 (protein kinase AMP-activated non-catalytic subunit beta 2) and CDK4 (cyclin dependent kinase 4) were identified. PRKAB2, a multi-substrate enzyme activated during metabolic stress caused by exercise, or hypoxia, is involved in regulating energy balance, glycogen metabolism and glycolysis [42], and is usually highly expressed in tumors [43]. CDK4 plays key roles in cell proliferation via driving the progression of cells into the DNA synthetic phase of the cell-division cycle [44], and is involved in the pathogenesis and inducing chemotherapy resistance in NPC [45, 46]. Whether TET2 inhibiting NPC progression is also associated with PRKAB2/CDK4 needs to be explored in the future studies.

## Conclusion

Our group demonstrates TET2 delays nasopharyngeal carcinoma progression by interacting with PKM and suppressing glycolysis, suggesting that TET2 may serve as a new therapeutic target for NPC.

## Supplementary information

Additional file 1: **Table S1**. Proteins potentially interacted with TET2 identified by mass spectrometry.

## Data Availability

The data in this study are available from the author for correspondence upon reasonable request.

## Abbreviations

NPC: Nasopharyngeal carcinoma
TET2: Ten-eleven translocation protein 2
mC: 5-Methylcytosine
5-hmC: 5-Hydroxymethylcytosine
PKM: Pyruvate kinase, muscle
HK: Hexokinase
PFK: Phosphofructokinase
PK: Pyruvate kinase
LDH: Lactate dehydrogenase
DMOG: Dimethyloxallyl glycine
H3K4me3: Histone H3K4 trimethylation
CHIP: Chromatin immunoprecipitation
